# Comparative Analysis Using Raman Spectroscopy of the Cellular Constituents of *Lacticaseibacillus paracasei* Zhang in a Normal and Viable but Nonculturable State

**DOI:** 10.3390/microorganisms11051266

**Published:** 2023-05-11

**Authors:** Qiuhua Bao, Xiaoyu Bo, Lu Chen, Yan Ren, Huiying Wang, Lai-Yu Kwok, Wenjun Liu

**Affiliations:** 1 Key Laboratory of Dairy Biotechnology and Engineering, Ministry of Education, Inner Mongolia Agricultural University, Hohhot 010018, China; nmgbqh@126.com (Q.B.); bxy1610@163.com (X.B.); 13154885560@163.com (L.C.); m15049697861@163.com (H.W.); 2 Key Laboratory of Dairy Products Processing, Ministry of Agriculture and Rural Affairs, Inner Mongolia Agricultural University, Hohhot 010018, China; 3 Inner Mongolia Key Laboratory of Dairy Biotechnology and Engineering, Inner Mongolia Agricultural University, Hohhot 010018, China; 4 School of Life Science and Technology, Inner Mongolia University of Science and Technology, Baotou 014016, China; renyanxq@163.com

**Keywords:** *Lacticaseibacillus paracasei* Zhang, viable but nonculturable (VBNC) state, induction, molecular composition, single-cell Raman spectroscopy

## Abstract

This study aimed to investigate the molecular composition of a viable but nonculturable (VBNC) state of a probiotic strain, *Lacticaseibacillus paracasei* Zhang (*L. paracasei* Zhang), using single-cell Raman spectroscopy (SCRS). Fluorescent microcopy with live/dead cell staining (propidium iodide and SYTO 9), plate counting, and scanning electron microscopy were used in combination to observe bacteria in an induced VBNC state. We induced the VBNC state by incubating the cells in de Man, Rogosa, and Sharpe broth (MRS) at 4 °C. Cells were sampled for subsequent analyses before VBNC induction, during it, and up to 220 days afterwards. We found that, after cold incubation for 220 days, the viable plate count was zero, but active cells could still be observed (as green fluorescent cells) under a fluorescence microscope, indicating that *Lacticaseibacillus paracasei* Zhang entered the VBNC state under these conditions. Scanning electron microscopy revealed the altered ultra-morphology of the VBNC cells, characterized by a shortened cell length and a wrinkled cell surface. Principal component analysis of the Raman spectra profiles revealed obvious differences in the intracellular biochemical constituents between normal and VBNC cells. Comparative analysis of the Raman spectra identified 12 main differential peaks between normal and VBNC cells, corresponding to carbohydrates, lipids, nucleic acids, and proteins. Our results suggested that there were obvious cellular structural intracellular macromolecular differences between normal and VBNC cells. During the induction of the VBNC state, the relative contents of carbohydrates (such as fructose), saturated fatty acids (such as palmitic acid), nucleic acid constituents, and some amino acids changed obviously, which could constitute a bacterial adaptive mechanism against adverse environmental conditions. Our study provides a theoretical basis for revealing the formation mechanism of a VBNC state in lactic acid bacteria.

## 1. Introduction

*Lacticaseibacillus paracasei* Zhang (*L. paracasei* Zhang, formerly named *Lactobacillus casei* Zhang) is a lactic acid bacterium isolated from traditional fermented mare milk in Inner Mongolia, China [1,2,3]. This bacterium has been extensively studied and was the first probiotic bacterium to have its genome completely sequenced in China [2,3]. It shows good probiotic properties. The *L. paracasei* Zhang strain has good acid resistance and bile salt resistance, strong abilities in immune regulation and antioxidation, and has been widely commercially used as a probiotic food additive and supplement [4]. Lactic acid bacteria are frequently added during food production, and some of them enter the viable but nonculturable (VBNC) state to maximize their survival when facing unfavorable growth conditions during food processing and fermentation [5,6]. However, the current knowledge of VBNC bacteria has mainly been developed in studies of pathogenic bacteria, and our understanding of the VBNC state of probiotics or bacteria that are used in food is limited [7].

The VBNC state was first reported in 1982 based on studies of *Escherichia coli* and *Vibrio cholerae* in estuaries and marine environments [8]. The bacterial VBNC state is highly similar to dormancy, whereby the bacteria cannot grow in standard media but survive under low metabolic activity and gene expression [8,9]. Adverse environments, such as radiation, low temperatures, starvation, and antibiotic use, can induce the bacterial VBNC state [10,11]. To date, more than 100 pathogenic and non-pathogenic bacteria have been reported to enter the VBNC state, and most studies on VBNC cells have focused on pathogenic bacteria [12,13,14]. Normal cells and VBNC cells exhibit obvious differences in their morphology and physiology, as well as compositional differences in their cell walls, cell membranes, and intramolecular content [15,16]. Thus, it should be possible to distinguish between normal healthy cells and VBNC cells. Owing to the low activity of VBNC bacteria, they cannot be cultivated easily in conventional media; this creates the risk of false negatives if conventional bacterial detection methods (e.g., plate count) are used for detection or cell recovery [17]. Therefore, it is of interest to explore the formation mechanism of the VBNC state in order to further understand the biology of these cells, which can then inform subsequent studies.

**Figure 1:**
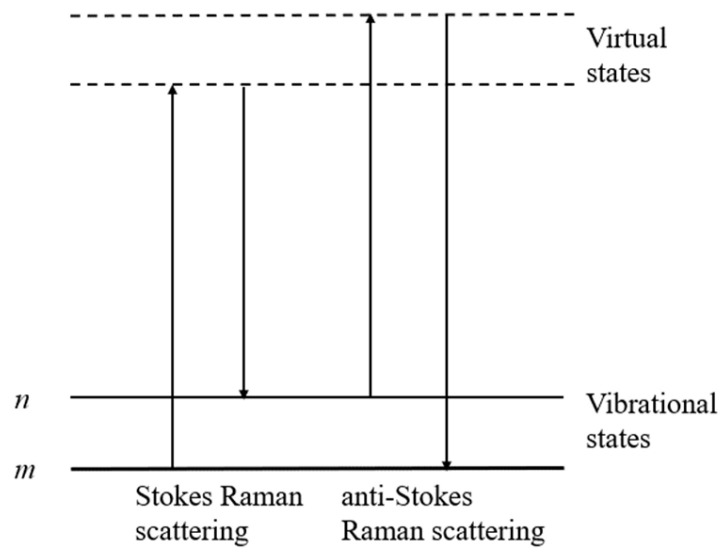
Illustration of the Raman scattering process. The lowest-energy vibrational state *m* is shown at the foot of the image, with states of a higher-energy excited vibrational state (*n*) above it. Both the low energy (upward arrows) and the scattered energy (downward arrows) have higher energy levels than the energy of a vibration.

The current methods for detecting VBNC cells include fluorescence microscopy coupled with staining kits, molecular detection techniques, and flow cytometry [12,18]. Cells are basic units of bacterial activity and organisms’ morphological structures. In fact, the physiological state of a cell culture population is not always synchronized, as is evidenced by the intra-population heterogeneity of cells [19]. Thus, single-cell-level research has been developed to produce more accurate and comprehensive information, reflecting real-time physiological states and cellular processes [20]. However, single-cell-level analysis is often limited by the availability of minute amounts of cellular material, hindering the detection sensitivity [21]. Single-cell Raman spectroscopy (SCRS) is a powerful and cost-effective method that has emerged in recent years for profiling the intracellular molecular composition (such as DNA, RNA, proteins, and lipids) of single cells [22]. The sample is illuminated by a monochromatic laser in the Raman spectrometer. The molecules in the sample interact with the excited light, scattering the light with the vibrations of the molecular bonds. A small fraction of scattered photons has a different frequency from the incident laser. The outcoming scattered light can be a photon with a lower frequency than the original photon (known as Stokes Raman scattering) or with a higher frequency (known as anti-Stokes Raman scattering) [23]. The Raman scattering process is shown in Figure 1. Profiles generated by SCRS provide rich biomolecular information, including the spectral vibration information of the biological bonds of nucleic acids, proteins, carbohydrates, and lipids, which can define and identify different cell types and physiological states. Moreover, cells analyzed by SCRS are not labeled, contain no specific markers, and can be from any complex cell population [24]. This approach has also been used in quantitatively distinguishing between bacterial species [25], measuring the general metabolic activity of cells, probing for a specific metabolite [26], and analyzing the catabolism of cell activity [27]. The SCRS approach is generally accurate, and Raman spectra can be obtained rapidly at the sub-second level [28,29]. Therefore, SCRS application is of great significance for exploring microbes at the single-cell level.

More than 95% of microorganisms in nature are in a VBNC state due to various environmental pressures. They cannot be isolated and cultured easily, leading to inadequate research on and utilization of these microbes [30]. In addition, current studies on VBNC bacteria mostly focus on induction conditions, detection methods, and the resuscitation environment, but lack in-depth analysis of their bacterial activity characteristics [31]. Therefore, this study used SCRS to explore the VBNC state of a well-studied food-use and probiotic bacterium, namely *L. paracasei* Zhang, to investigate its changes in intracellular macromolecules during the induction of a VBNC state. This study aimed to gain a better understanding of the VBNC state of lactic acid bacteria and provide a theoretical basis for revealing the formation mechanisms of a VBNC state in lactic acid bacteria.

## 2. Materials and Methods

### 2.1. Bacterial Strain

The probiotic strain *L. paracasei* Zhang was obtained from the Key Laboratory of Dairy Biotechnology and Engineering of Inner Mongolia Agricultural University, Ministry of Education, Lactic Acid Bacteria Culture Collection (LABCC). The strain was originally isolated from koumiss, a traditionally fermented mare milk product from Inner Mongolia, and its taxonomic identity was confirmed using biochemical, physiological, and molecular methods [1]. The bacterial strain was preserved as a glycerol stock and was stored at −80 °C.

### 2.2. Cell Culture and Induction of VBNC State

A pure culture of *L. paracasei* Zhang was grown from the stock culture in 5 mL of de Man, Rogosa, and Sharpe (MRS) broth (Oxoid, Basingstoke, UK) at 37 °C for 18 to 22 h without shaking. A logarithmic phase culture of *L. paracasei* Zhang was washed twice in sterile saline (0.85% NaCl solution), resuspend in sterile MRS broth, and diluted to 10^7^ cells/mL in saline to induce the VBNC state at various temperatures (Table 1). The cells were allowed to stand at these specific conditions for an extended period (until no viability was detected by the plate count) for the induction of the VBNC state. Aliquots of cell samples were collected for analysis at the start of the experiment, every 10 days for the first 120 days (until no viability was detected), and regularly the 220th day of induction under condition A.

### 2.3. Determining VBNC State by Plate Counting, Bacterial Viability Staining, and Cell Morphology Analysis

The bacterial culture was inoculated into the appropriate medium for induction of the VBNC state, and the inoculated induction cultures were mixed. Half of a milliliter of each mixed induction culture was serially diluted by a tenfold gradient and plate counted on MRS agar after 48 h of incubation at 37 °C. The number of colonies grown on the plates was recorded. The cell viability was also determined using a LIVE/DEAD BacLight Bacterial Viability Kit L7012 (based on the stains, SYTO 9, and propidium iodide) [32]. Briefly, a volume of 2 μL mix (1.5 μL each of SYTO 9 and propidium iodide dissolved in 100 μL of dimethyl sulfoxide) was added to 50 μL of the cell suspension, which had previously been washed twice with sterile saline NaCl (0.85%) solution. After 15 min staining at 25 °C, 2 μL of this suspension was fixed on microscope slides for observation on a Lecia DM4000B fluorescent microscope (under 1000× magnification; Leica Microsystems, Wetzlar, Germany).

The *L. paracasei* Zhang cells were considered to have entered a complete VBNC state when the number of cells (per milliliter) determined by plate counting (i.e., the number of colony-forming units/mL, CFU/mL) was zero, while living cells (as green florescent cells) were still detected when observed under a fluorescence microscope (red fluorescence represented dead cells). The cell morphology was documented using scanning electron microscopy. For scanning electron microscopy, bacterial cultures were centrifuged at 1000× *g* at 4 °C for 10 min. The bacterial cells were washed 3 times with 10 mM phosphate buffered saline at 1000× *g* for 10 min. Next, the cells were fixed in 3% glutaraldehyde for more than 2 h, followed by washing 3 times with phosphate buffered saline and dehydration through the 10%, 30%, 50%, 70%, 80%, 90%, and 100% ethanol series. The dehydrated cells were dried by carbon dioxide under a critical point, then mounted, gold-coated, observed, and photographed with a scanning electron microscope (SU8010, Hitachi Ltd., Tokyo, Japan) [33].

### 2.4. Single-Cell Raman Spectroscopy

Healthy and VBNC cells were subjected to SCRS. Healthy cells were prepared by culturing the bacteria in MRS medium at 37 °C for 18 h when the culture entered the logarithmic growth phase. Among the 6 induction conditions, only cultures that were induced in MRS broth at 4 °C were found to enter the VBNC state and were thus further investigated by SCRS. Cell aliquots were collected at 4 time points (in triplicate) after starting VBNC induction (i.e., 0, 120, 180, and 220 d), and the cells were centrifuged (5200 rpm × 3 min) at room temperature. Cells were washed three times with deionized water to remove the culture medium. Each sample was resuspended and transferred (1.5 μL) to a calcium fluoride microscope slide, and then air-dried for SCRS analysis. Raman spectra were obtained using a modified confocal Raman-fluorescent microscope (LabRam HR system, Horiba Ltd., Stanmore, UK) according to Wang et al. [34]. Signals were read with a 100× magnification dry objective (NA = 0.90; Olympus, London, UK). A 100 mW × 532 nm Nd: YAG laser (Ventus, Laser Quantum Ltd., Cheshire, UK) was used as the light source, and the power of the laser beam was maintained between 2 and 5 mW. The acquisition time for each spectrum was 10 s, and single-cell Raman spectra of 20 cells were collected from the triplicate sample (i.e., n = 60 spectra per sampling time point). Scattered photons were collected by a Newton EMCCD detector (DU970N-BV, Andor, Belfast, UK). The SCRS was performed at the Qingdao Institute of Bioenergy and Bioprocess Technology, Qingdao, China.

### 2.5. Pre-Processing of Raman Spectra

Raw Raman spectra were pre-processed with the spectral software suite LabSpec 5 (HORIBA Scientific, Orsay, France), including background subtraction and baseline correction by a second-degree polynomial algorithm; then, zero and area normalization were obtained [35]. For each sample, the background spectrum provided for subtraction was generated as the average of five spectra acquired from the slide around the cell. The drawing of spectral maps and statistical analysis of spectral data were carried out in Origin 2021. The biochemical fingerprint range (600 to 1800 cm^−1^) was extracted for subsequent analysis and obtaining useful information in the Raman bands [36].

### 2.6. Principal Component Analysis (PCA)

Normalized fingerprint ranges of the SCRS of normal and VBNC-state *L. paracasei* Zhang cells were subjected to PCA, which was performed with Matlab R2016a [35]. In PCA, the dimension of the dataset was reduced; and only the first 30 principal components were retained in our analysis. The number of principal components in the PCA was determined based on the variability that could explain more than 70% of the data.

## 3. Results and Discussion

### 3.1. Cell Activity of L. paracasei Zhang in a VBNC State

**Figure 2:**
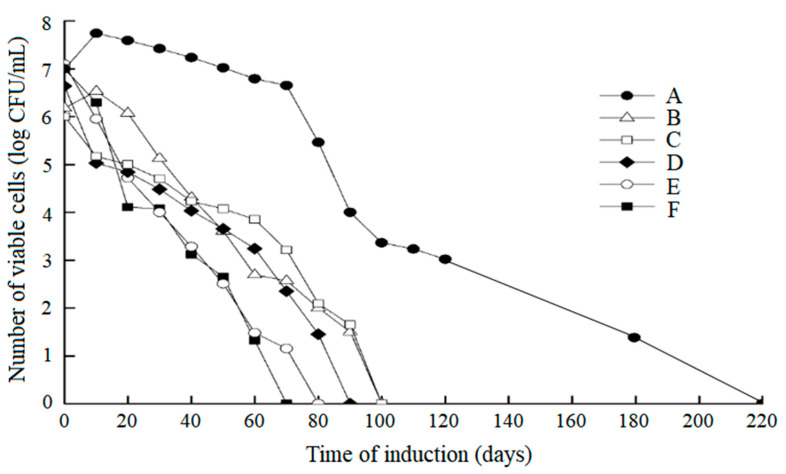
Induction of viable but nonculturable cells (VBNC) of *Lacticaseibacillus paracasei* Zhang (*L. paracasei* Zhang) under different growth conditions. (A) MRS broth, 4 °C. (B) Luria–Bertani broth, 4 °C, pH 2.0–3.0. (C) Sterilized distilled water, room temperature. (D) Sterilized distilled water, 4 °C. (E) 0.85% NaCl solution, 4 °C. (F) 0.85% NaCl solution, −20 °C.

**Figure 3:**
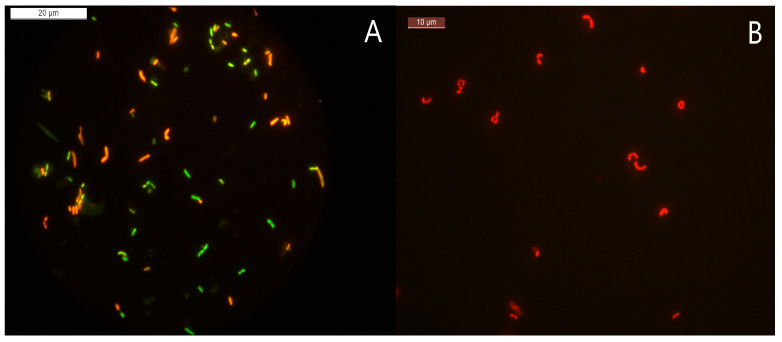
Detection of cell viability using a LIVE/DEAD BacLight Bacterial Viability Kit. The kit distinguishes live and dead cells via the action of two dyes, i.e., SYTO 9 and propidium iodide. Dead or dying cells are stained red due to the entrance of propidium iodide through cell membranes with compromised integrity. Cells with an intact membrane will stain green. (**A**) Viable but nonculturable bacteria. (**B**) Dead bacteria.

The probiotic strain *L. paracasei* Zhang was induced under different conditions (Table 1). During the induction process, the number of viable cells and the bacterial cell activity were monitored using the plate count and fluorescent and scanning electron microscopy. The number of viable cells decreased with time, as expected (Figure 2). The loss of viability of the cells under condition A was much slower than under the other conditions (up to 220 days under condition A versus 70 to 100 days under the other conditions). Meanwhile, the integrated results of the plate count and fluorescent microscopic counting of live and dead cells showed that only condition A (MRS broth, 4 °C) was able to induce the complete cell population to enter the VBNC state. Under growth condition A, unculturable (did not grow in the culture medium) but active bacteria (stained green fluorescent by the LIVE/DEAD BacLight Bacterial Viability Kit in Figure 3A) could still be observed after 220 days of the bacterial induction, indicating that the bacteria entered the VBNC state [37]. Under other growth conditions, when the number of viable bacteria was zero, all bacteria were red and fluorescent after staining with the LIVE/DEAD BacLight Bacterial Viability Kit (Figure 3B), indicating that all of the cells died. Many studies report that low temperature, as a stressor, can induce bacteria to enter the VBNC state [38]. Thus, only cells cultured under condition A were further analyzed by SCRS.

**Figure 4:**
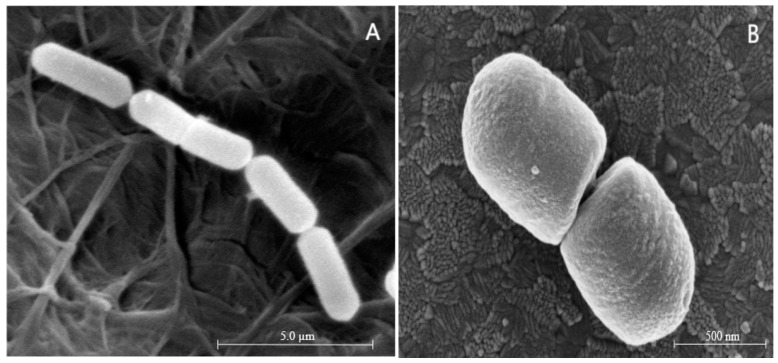
Scanning electron micrographs of *Lacticaseibacillus paracasei* Zhang. (**A**) Normal cells. (**B**) Viable but nonculturable cells.

Meanwhile, the morphology of healthy and VBNC *L. paracasei* Zhang cells collected before and after induction of the VBNC state was observed using a scanning electron microscope (Figure 4). The cell morphology of healthy *L. paracasei* Zhang cells appeared as short rods, and the average length of single bacteria was about 2.02 μm (Figure 4A). The ultra-morphology of VBNC *L. paracasei* Zhang had obviously changed, and was characterized by a significantly shortened cell length and wrinkled and rough cell surfaces (Figure 4B). The morphological change in the VBNC cells is considered an adaptive response to protect the cells against harsh environmental conditions [39]. Reducing their size allows cells to have the maximum nutrient absorption surface while maintaining the minimum cell mass, which may be a general mechanism for coping with environmental stress rather than a specific characteristic of VBNC cells. It is likely that VBNC cells may exhibit some degree of change in shape, but cells with altered shapes may not necessarily be in a VBNC state [40].

### 3.2. Single-Cell Raman Spectroscopy for Discrimination between Different Induction Stages of L. paracasei Zhang

**Figure 5:**
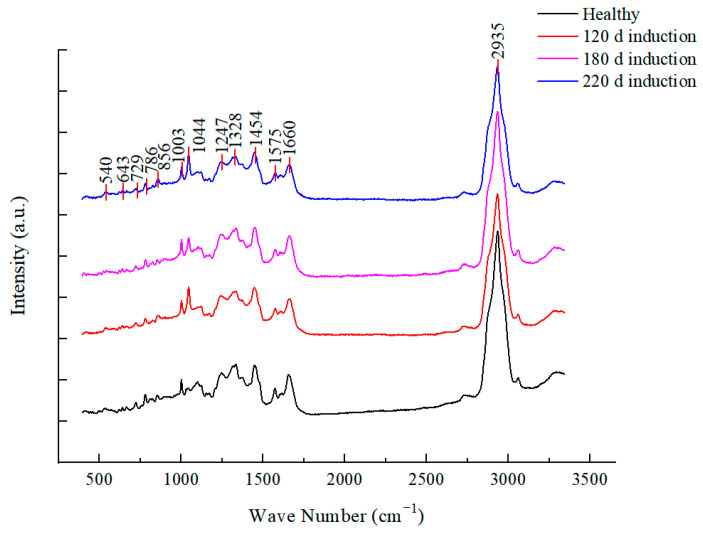
Averaged Raman spectra of *Lacticaseibacillus paracasei* Zhang at different induction stages.

**Figure 6:**
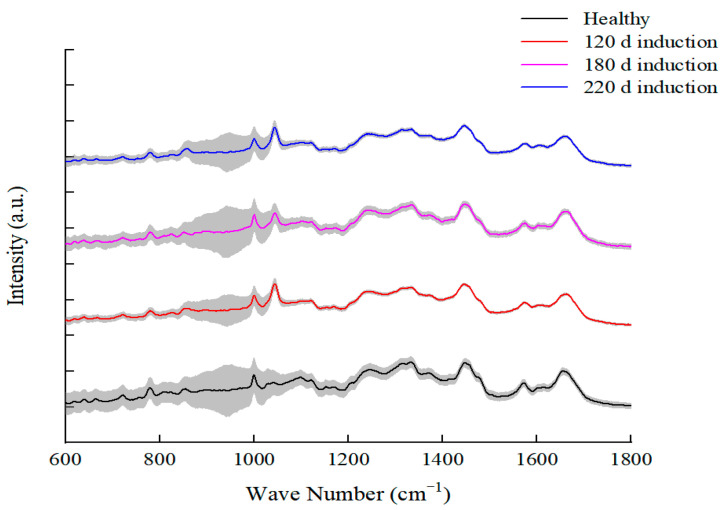
Mean Raman spectra of *Lacticaseibacillus paracasei* Zhang at different induction stages. The grey area represents the standard deviation (±1 standard deviation).

In this experiment, healthy (bacteria in logarithmic growth phase, cultured at 37 °C for 18 h) bacteria and *L. paracasei* Zhang incubated in MRS medium for 120, 180, and 220 days at 4 °C were collected for SCRS analysis. The average Raman spectra obtained after data processing are shown in Figure 5. The Raman spectrum as a whole reflects the cell composition and the structure of the intracellular macromolecules. The main characteristic peaks of the Raman spectra of *L. paracasei* Zhang cells were attributed to carbohydrates (885, 1071, and 1112 cm^−1^ peaks), while other signal peaks were attributed to lipids (860, 908, 1044 cm^−1^ peaks), proteins (883, 950, and 1003 cm^−1^ peaks), nucleic acid (729 cm^−1^, 786 cm^−1^, and 1092 cm^−1^ peaks), and amino acids (540 cm^−1^, 643 cm^−1^, and 1328 cm^−1^ peaks) (Table 2). The fingerprint region between 600 and 1800 cm^−1^ was concentrated (Figure 5), so the fingerprint peaks in this range were selected in subsequent analysis (Figure 6).

### 3.3. Principal Component Analysis of Raman Spectra of Normal and VBNC L. paracasei Zhang Cells

**Figure 7:**
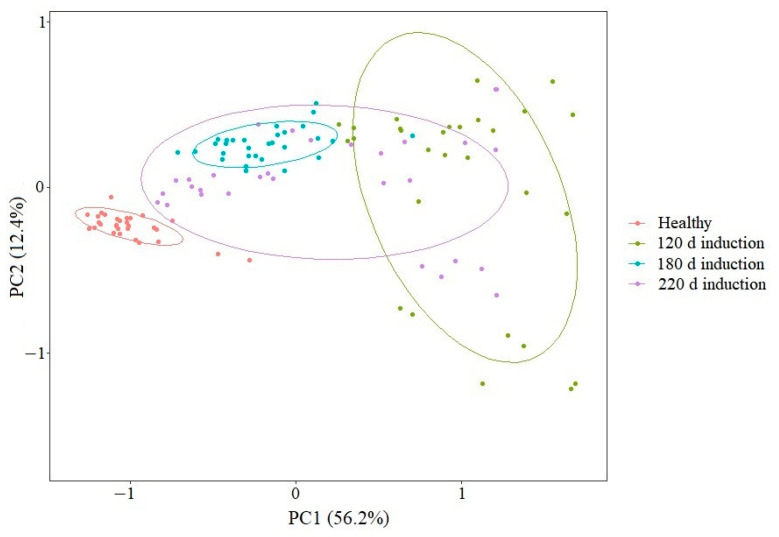
Principal component analysis of healthy and viable but nonculturable (VBNC) cells that underwent incubation for 120, 180, and 220 days.

The raman spectrum data of normal healthy cells (collected at logarithmic growth phase) and VBNC cells of *L. paracasei* Zhang (cultured at MRS broth at 4 °C for 120, 180, and 220 days) were analyzed using PCA (Figure 7). On the PCA score plot, the symbols representing healthy cells formed a tight cluster in the left upper quadrant, while the symbols representing the early and middle stages (days 120 and 180) of the induction of the VBNC state formed looser clusters in the plot; symbols representing the latest time point of VBNC induction (i.e., day 220) formed a tight cluster. The fact that the clusters of symbols representing healthy and VBNC cells did not overlap in the score plot shows that the Raman spectrum profiles of healthy and VBNC cells were obviously different. Furthermore, although there was some overlapping between the symbols of the different time points of VBNC induction, those representing the earlier time point of induction (i.e., day 120) were located away from the tight cluster of symbols representing the late time point (i.e., day 220). Notably, symbols representing some of the cells after 180 days of induction were also located close to the cluster of cells induced for 220 days. These results suggest that the VBNC induction was a long and gradual process of shifting of cell metabolic activity toward a dormant and inactive state, evidenced by the shift from the overall intra-time point heterogeneity of the Raman spectrum features of individual cells during (days 120 and 180) the VBNC process until reaching the end stage (day 220), when the Raman spectrum profile of the cells became homogenous again, as observed in the healthy cells.

**Figure 8:**
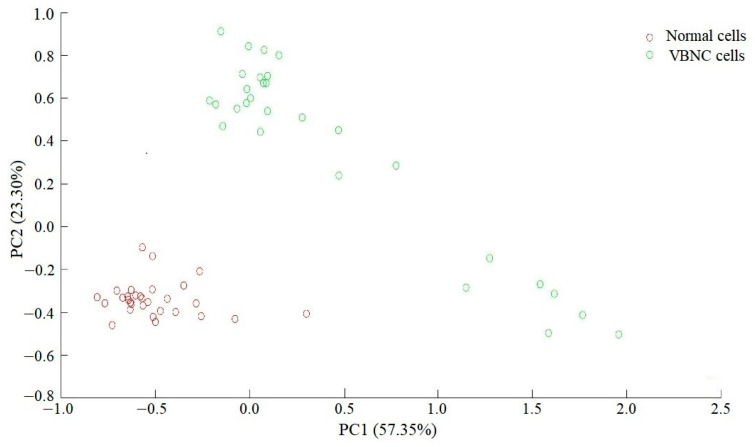
Principal component analysis of normal and viable but nonculturable (VBNC) *Lacticaseibacillus paracasei* Zhang cells.

The overall difference in the Raman spectra between the normal and VBNC states of *L. paracasei* Zhang was analyzed using PCA analysis. Symbols representing cells in the two different states were distributed in distinct clusters on the PCA score plot, suggesting that there were apparent cellular and metabolic differences (Figure 8).

### 3.4. Differences in Raman Spectra between Normal and VBNC L. paracasei Zhang

**Figure 9:**
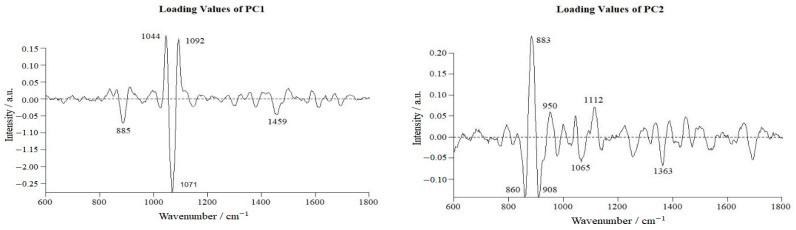
Loadings of principal components PC1 and PC2.

To identify the major Raman spectra features that distinguish normal cells from VBNC cells, we plotted the loadings of PC1 and PC2 and Raman spectral peaks with an absolute value of load greater than 0.05 (Figure 9). For PC1, the greatest Raman peaks were 885, 1044, 1071, 1092, and 1459 cm^−1^, while the greatest Raman peaks of PC2 were 883, 860, 908, 950, 1065, 1112, and 1363, cm^−1^. Comparative analysis of the SCRS profiles revealed the key differential metabolites between the different cell states. These twelve characteristic peaks were mapped to carbohydrates (three peaks), lipids (four peaks), nucleic acids (three peaks), and proteins (two peaks) (Table 2). We used these peaks as simple parameters to analyze the intracellular macromolecular changes in *L. paracasei* Zhang during induction.

### 3.5. Changes in Molecular Constituents of L. paracasei Zhang after VBNC Induction

**Figure 10:**
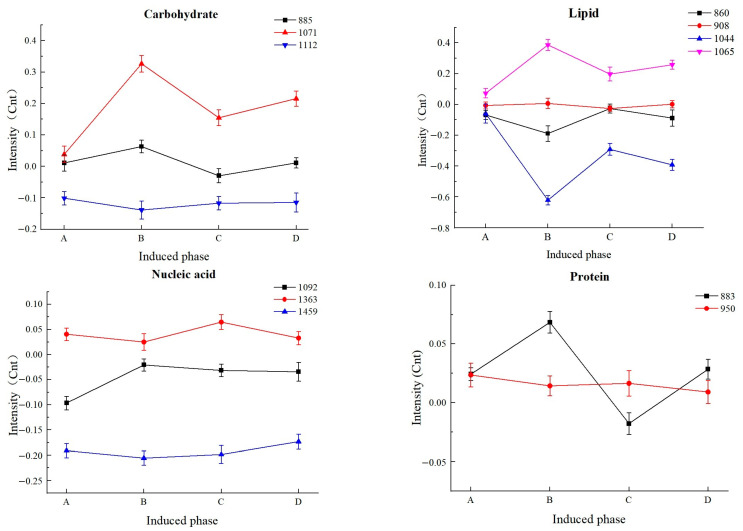
Changes in the signal intensity of intracellular constituent peaks during different induction stages of *Lacticaseibacillus paracasei* Zhang. The numbers specified in each plot represent the Raman frequency of each peak. (A) Healthy. (B) 120 days of induction. (C) 180 days of induction. (D) 220 days of induction.

Differences in the molecular constituents (carbohydrates, lipids, nucleic acids, and proteins) of healthy and VBNC *L. paracasei* Zhang cells at different induction periods are shown in Figure 10.

Carbohydrates are the most important constituents for discriminating between cell states, and they are the energy source used to maintain cell activity [44]. The variation in Raman peaks attributed to carbohydrates was analyzed. The intensity of the 1071 cm^−1^ peak (corresponding to fructose) increased first and then decreased, with large fluctuations. Smaller fluctuations were observed in the 885 cm^−1^ and 1112 cm^−1^ peaks. The contents of most carbohydrate-related constituents increased significantly during the early stage of induction, which could be an adaptive response towards the adverse environment. Then, carbohydrates were gradually consumed and depleted. After the complete cell population entered the VBNC state, the carbohydrate content increased slightly; this was likely generated intracellularly to provide a minimal level of energy for survival. Previous reports found that several genes involved in carbohydrate and energy metabolism in yeast and bacteria were downregulated in the VBNC state [12,45]. However, different stress conditions and microbial species exhibited different changes in their carbohydrate metabolism [44].

The patterns of changes in the intensity of peaks of 860 cm^−1^ (corresponding to *ν*_3_PO_4_^3−^ group in phosphoric acid) and 1044 cm^−1^ (corresponding to a phospholipid group) were similar during the various VBNC induction stages, while the 1044 cm^−1^ peak showed greater variation between the different stages of VBNC induction. These molecular groups are important intermediates in lipid synthesis and decomposition. The intensity of the 908 cm^−1^ peaks (corresponding to myristic acid) exhibited only minor fluctuations during the VBNC induction stage, not showing obvious differences from healthy cells. The peak of 1065 cm^−1^ was attributed to palmitic acid, and its signal intensity increased first and then decreased. In the study of Yoon et al. [39], VBNC *V. parahaemolyticus* ATCC 17802 induced at 4 °C for 80 days had increased amounts of saturated fatty acids, such as lauric acid (C12), myristic acid, palmitic acid (C16), and stearic acid (C18), whereas the quantity of unsaturated fatty acids was reduced. Palmitic acid is involved in enhancing the tolerance of *Vibrio parahaemolyticus* to low pH, ethanol, and NaCl [39,46]. The results indicated that bacterial cells in a VBNC state could alter their membrane fatty acid composition to cope with adverse environmental conditions.

Then, changes in Raman peaks of nucleic acids during VBNC induction were analyzed. The intensity of the 1459 cm^−1^ peak fluctuated slightly in the induction process, while the intensity of the 1363 cm^−1^ and 1092 cm^−1^ peaks fluctuated to varying degrees during late and early induction, respectively. The 1459 cm^−1^, 1363 cm^−1^, and 1092 cm^−1^ peaks were assigned to deoxyribose in nucleic acid, guanine, and phosphoric acid, respectively. These three chemical structures are the main constituents of nucleic acids. Overall, the fluctuation in the Raman peak intensity of nucleic acids is relatively small, indicating a reduction in nucleotide synthesis activity in VBNC bacteria, which would possibly interfere with DNA replication and cell growth suspension. Indeed, most energy-dependent nucleotide biosynthesis pathways were found to be turned off after the bacteria entered the VBNC state [47,48].

Changes in the intracellular protein-related constituents during induction were then analyzed. The intensity of the 883 cm^−1^ peak fluctuated significantly, while a smaller fluctuation was seen in the 950 cm^−1^ peak. The content of the ρ(CH_2_) group (corresponding to the 883 cm^−1^ peak) increased first, followed by a decrease during VBNC induction, then increased again at the end stage of VBNC induction. The Raman signal intensity of the single bond stretching vibration (corresponding to the 950 cm^−1^ peak) of the amino acids proline and valine showed only small variations during VBNC induction, but their expression decreased with the induction time. This might be a result of the continuous consumption of amino acids to support bacterial growth and survival. Postnikova et al. [49] found that many genes involved in amino acid metabolism were upregulated, indicating that modulating the biosynthesis and degradation of amino acids is necessary for transitioning to the VBNC state.

Food-use bacteria often face extensive environmental stress during the food production process, and some of them may enter the VBNC state. However, few studies have systematically elucidated the physiology of the VBNC state of food-use or probiotic bacteria. Thus, this study investigated the metabolic properties of VBNC *L. paracasei* Zhang, a probiotic strain, using SCRS. Single-cell Raman spectroscopy is a powerful method for monitoring intracellular macromolecular changes, but has seldom been used for analyzing changes in the bacterial VBNC state. A previous study analyzed the metabolic changes in chlorine-stress-induced VBNC *Pseudomonas aeruginosa*, a potential pathogen [48]. The commonality between our study and the work of Qi and Liu (2022) [48] is that both studies analyzed the metabolic properties of VBNC bacteria using SCRS, focusing on four major groups of macromolecules, including carbohydrates, fatty acids, nucleic acids, and proteins. Qi and Liu (2022) [48] found that the metabolism of nucleotides, amino acids, and central carbon in VBNC bacteria decreased substantially, which is consistent with our data. However, Qi and Liu (2022) [48] observed that the unsaturated fatty acid biosynthesis increased in VBNC bacteria; this contrasts with our data, which reveal the elevation of the content of a saturated fatty acid (palmitic acid) but not unsaturated fatty acids. The discrepant results could be due to the basic metabolic and cell structural differences between the two bacterial species. Since *L. paracasei* is a Gram-positive bacterium, and *Pseudomonas aeruginosa* is a Gram-negative bacterium, it would not be surprising if the two bacteria underwent different metabolic changes when tranitioning into a dormant VBNC state. The work of Qi and Liu (2022) [48] identified nine differential metabolic markers (including pyruvate, glyoxylate, guanine, glutamate, sn glycero-3-phos-phocholine, fatty acid, d-alanine, glutathione, and N-Butanoyl-d-homoserine lactone) between normal and VBNC cells. Although only one common differential intracellular constituent (namely, guanine) was identified in the two studies, both works consistently found that VBNC bacteria maintain low metabolic activity and exhibit obvious intrapopulation-level metabolic heterogeneity.

## 4. Conclusions

This study investigated the VBNC state of lactic acid bacteria using a well-studied probiotic bacterium, namely *L. paracasei* Zhang, as a model. The Raman spectrum analysis found obvious differences in the levels of various intracellular constituents (including fructose, phospholipid acid, palmitic acid, phosphoric acid, and guanine) between normal and VBNC cells, which are useful in distinguishing between cell states. Raman spectroscopy generates intracellular macromolecular profiles without any fixation or labelling, thereby allowing the monitoring of live single cells without sacrificing their viability or causing any physiological damage. In short, our study explored the VBNC state of lactic acid bacteria using Raman spectroscopy. The current study provides a theoretical basis for revealing the formation mechanism of the VBNC state in lactic acid bacteria; it also demonstrates the usefulness of Raman spectroscopy in detecting macromolecules at the single-cell level without any labels.

## Figures and Tables

**Table 1:** Conditions for inducing a viable but nonculturable (VBNC) state.

Condition	VBNC Induction Medium	pH	Temperature
A	MRS broth	6.8	4 °C
B	Luria–Bertani broth	2.0 to 3.0	4 °C
C	Sterilized distilled water	7.0	Room temperature
D	Sterilized distilled water	7.0	4 °C
E	0.85% NaCl solution	7.0	4 °C
F	0.85% NaCl solution	7.0	−20 °C

**Table 2:** Molecular assignment of main Raman peaks of *Lacticaseibacillus paracasei* Zhang.

Molecular Group	Raman Frequency (cm^−1^)	Chemical Assignment	Reference
Carbohydrate	885	Disaccharide (cellobiose), (C-O-C) skeletal mode	[41]
	1071	D-Fructose-6	[42]
	1112	Saccharide band (overlaps with acyl band)	[41]
Lipid	860	Phosphate group; phosphatidic acid	[41]
	908	Myristic acid	[42]
	1044	*ν*_3_PO_4_^3−^ (symmetric stretching vibration of *ν*_3_PO_4_^3−^ of HA)	[41]
	1065	Palmitic acid, fatty acid	[41]
Protein	883	ρ (CH_2_) (protein assignment)	[41]
	950	Most probably due to single bond stretching vibrations of the amino acids proline and valine and polysaccharides	[41]
	1003	Symmetric ring breathing mode of phenylalanine	[43]
Nucleic acid	729	Adenine ring breathing	[43]
	786	Cytosine	[35]
	1092	Phosphodioxy	[41]
	1363	Guanine (N_7_, B, Z-marker)	[41]
	1459	Deoxyribose; δ(CH_2_)	[41]
Others	540	L-Histidine	[42]
	643	Glutathione	[42]
	1247	Amide III	[43]
	1328	L-Tryptophan	[42]
	1575	Tryptophan-related bands	[27]
	1660	L-Valine	[42]
	2935	CH stretching of lipid and protein	[43]

## Data Availability

The datasets generated and/or analyzed during the course of this study are available from the corresponding author on reasonable request.
